# Intranasal Administration of Chitosan Against Influenza A (H7N9) Virus Infection in a Mouse Model

**DOI:** 10.1038/srep28729

**Published:** 2016-06-29

**Authors:** Mei Zheng, Di Qu, Haiming Wang, Zhiping Sun, Xueying Liu, Jianjun Chen, Changgui Li, Xuguang Li, Ze Chen

**Affiliations:** 1 Shanghai Institute of Biological Products, Shanghai 200052, China; 2 Biosafety Level-3 Laboratory, Key Laboratory of Medical Molecular Virology MOE & MOH, School of Basic Medical Sciences, Fudan University, Shanghai 200032, China; 3 State Key Laboratory of Virology, Wuhan Institute of Virology, Chinese Academy of Sciences, Wuhan 430071, Hubei, China; 4 National Institutes for Food and Drug Control and WHO Collaborating Center for Standardization and Evaluation of Biologicals, Beijing, China; 5 Centre for Vaccine Evaluation, Biologics and Genetic Therapies Directorate, HPFB, Health Canada, Ottawa, ON, Canada; 6 College of Life Sciences, Hunan Normal University, Changsha 410081, Hunan, China

## Abstract

Influenza virus evolves constantly in an unpredictable fashion, making it necessary to vaccinate people annually for effective prevention and control of influenza. In general, however, during the first wave of an influenza outbreak caused by a newly emerging virus strain, influenza morbidity and mortality have been observed to rise sharply due to the lack of a matching vaccine. This necessitates the exploration of novel intervention approaches, particularly those prophylactic or therapeutic agents that have a broad range of antiviral activities and are also proven to be non-toxic. Here, we reported that stimulation of the innate immune system by intranasal administration of chitosan as a single agent was sufficient to completely protect BALB/c mice from lethal infection by H7N9 virus, a newly emerged viral strain that is highly pathogenic to humans. Remarkably, animals could still be protected against lethal challenge by H7N9 (10×LD_50_), even ten days after the intranasal chitosan administration. The significantly enhanced infiltration of leukocytes in the bronchoalveolar lavage and elevated levels of proinflammatory cytokines in the bronchia/lung tissues revealed the potent activation of mucosal immune responses by intranasally delivered chitosan. We also observed that chitosan can protect mice from three other virus strains. The marked breadth and magnitude of protection against diverse viral strains makes chitosan an attractive candidate as a universal anti-influenza agent.

Influenza is an acute infectious disease of the respiratory tract and has high morbidity and mortality^1^. In March 2013, human infections with a new avian influenza A (H7N9) virus were reported in China^2^,^3^. Most of these cases are believed to result from exposure to infected poultry or contaminated environments. Although no evidence of sustained person-to-person spread of H7N9 has been found, there is some evidence pointing to limited person-to-person spread under specific circumstances^4^. The virus spread to many other places in China within a few months; as of today, infection of humans by this virus remains a serious concern^5^. Specifically, there were 246 fatal cases among the 670 confirmed human cases by the end of November, 2015^6^,^7^,^8^. There is no H7N9 vaccine available at this time, although some vaccine manufacturers have entered clinical evaluation of H7N9 vaccine^9^. In addition, the current anti-influenza treatments have been associated with the emergence of drug resistant viruses^10^,^11^,^12^,^13^,^14^. Given these findings, it is critically important to explore new therapeutic approaches.

While it is well established that neutralizing antibodies and cytotoxic T lymphocytes (CTL) largely contribute to specific immune responses against the influenza virus^15^,^16^, innate immunity also plays a significant role in host defenses against it. Specifically, the mucosal barrier is the first line of defense against respiratory infections. When a pathogen enters the mucosal layer, the innate immune cells residing in the layer first come into play to clear pathogens^17^,^18^,^19^,^20^. Indeed, activation of the innate immune system can substantially suppress the replication of the influenza virus in animal models. For example, intranasal administration of CT (cholera toxin), LT (heat-labile enterotoxin) and CpG (CpG-oligodeoxynueleotides, CpG-ODN) can protect mice from lethal virus challenges^21^.We previously reported that chitosan can function as an excellent adjuvant to improve the immunogenicity of M1- and M2-based candidate vaccines^22^,^23^. Here, we report that intranasal administration of chitosan alone could completely protect BALB/c mice from lethal H7N9 viral challenges, an observation that is also reproduced using three other subtypes of influenza viruses as challenging pathogens.

## Results

### Intranasal chitosan administration protected mice from lethal H7N9 virus challenge.

To investigate whether intranasal administration of chitosan in BALB/c mice could protect mice against H7N9 influenza virus infection, various doses of chitosan were given to mice via the intranasal (i.n.) or intraperitoneal route (i.p.) (Table 1). We also included PR8 [A/Puerto Rico/8/34 (H1N1)] in parallel as a control to determine whether there is any difference in the magnitude of protection. A total of 286 mice were randomly divided into 11 groups, with 26 mice in each group. Ten mice were monitored to observe the survival rate of the animals, while the rest of the animals were used for the analyses of virus loads in the lungs or cytokine. Following various dosing and administration schedules (see below for details), the mice were challenged i.n. with lethal doses of (10 × LD_50_) of H7N9 or PR8 and monitored for 21 days to determine survival rates.

**Figure 1:**
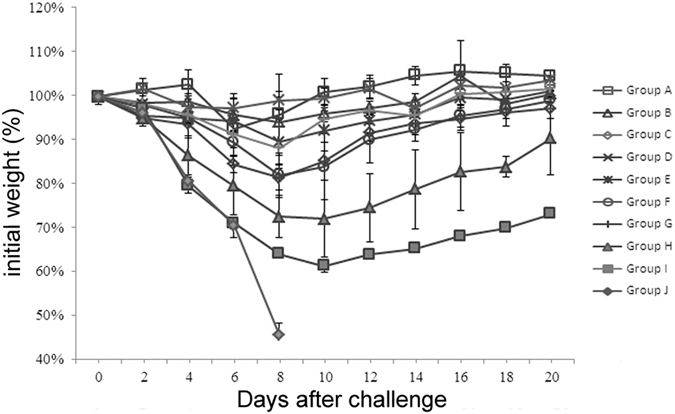
Measurements of the body weight of mice after H7N9 challenge. The dosing/challenging schedule in addition to the grouping of the animals were the same as described for Table 1. Specifically, mice were given 100 μg chitosan once or twice (i.n.) in group A–C or D–F and were challenged days 1, 3, 5 after the first or second chitosan administration. In group G and H, mice were dosed twice with 30 or 10 μg chitosan (i.n.) and were challenged 3 days after the second chitosan administration, respectively. In group I, mice were dosed twice with 100 μg chitosan via the intraperitoneal route and were subsequently challenged 3 days after the second chitosan administration. The naive mice were used as the control group (group J). The mice were challenged with a lethal dose of A/Shanghai/2/2013(H7N9) (10×LD_50_) and observed for 21 days to determine the protective effect.

As presented in Table 2, without regard to whether the animals were dosed once or twice with chitosan (Table 2, groups A–F), all of them were fully protected (100%). This is irrespective of whether the viral challenge was conducted 1, 3 or 5 days later. Moreover, the protection effect was found to be dose-dependent, as the survival rates were found to decrease with lower amounts of chitosan (30 μg or 10 μg) (Group G and H). Importantly, i.p. injection of chitosan protected only 10% of animals even when the highest dose was employed (100 μg) (Group I). As overall survival rates were monitored over 21 days, we determined the virus replication in mice three days after the H7N9 viral challenge; we found the viral loads in trachea/lung tissues from chitosan-treated mice were significantly lower than that from the control group J (p value of group A to E were <0.0001, 0.0116, 0.0260, 0.0001, and 0.0146, respectively) (p < 0.05). Finally, we also recorded the weight and clinical signs observed in these animals during the entire course. As shown in Fig. 1, mice challenged 1, 3, or 5 days after one or two chitosan administrations barely lost any body weight (Groups A–F). In sharp contrast, the untreated group and mice treated with chitosan by i.p. administration lost substantial body weight (Groups I and J), in addition to marked clinical signs (including piloerection, inactivity and huddling) observed within three days of the viral challenge. These results are largely in agreement with those obtained from survival studies (Table 2).

**Figure 2:**
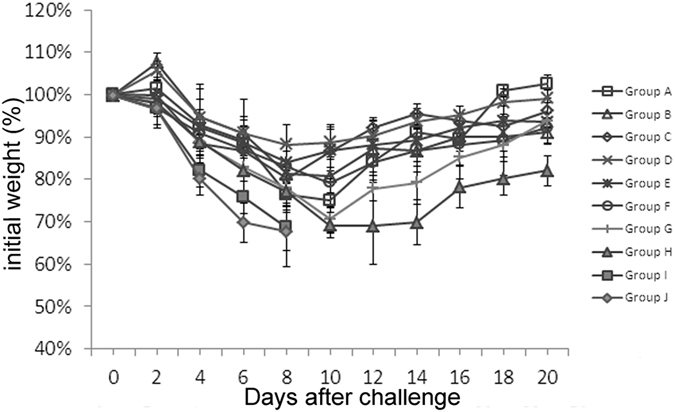
Measurements of the body weight of mice after PR8 challenge. Mice were treated with various dosing/challenging schedules and were grouped in the same way as presented in Table 1. The mice were challenged with a lethal dose of A/Puerto Rico/8/34 (H1N1) (10×LD_50_) on different days and observed for 21 days to determine the protective effect.

We next investigate whether chitosan could also protect the animals from infection with a different viral strain (PR8). As shown in Table 2, 60%, 80% and 70% survival rates were obtained when challenges were conducted 1, 3, or 5 days after chitosan injection. Moreover, if the animals were dosed twice, higher levels of protection were achieved (90%, 100% and 80%) (Group D–F). The data from virus titration in the BAL (bronchoalveolar lavage) (Table 2) and monitoring of body weight (Fig. 2) were both consistent with survival rates of the mice. While the protection against PR8 virus seems to be slightly less effective than that against H7N9 virus, these results are generally similar to those obtained from H7N9 experiments, i.e., two doses of chitosan at 100 μg could fully protect the 80-100% animals if the viral challenges were performed 1–5 days after the second injection; it is also clear that the i.n. route of administration was imperative.

### Duration of protective immune response induced by chitosan

**Figure 3:**
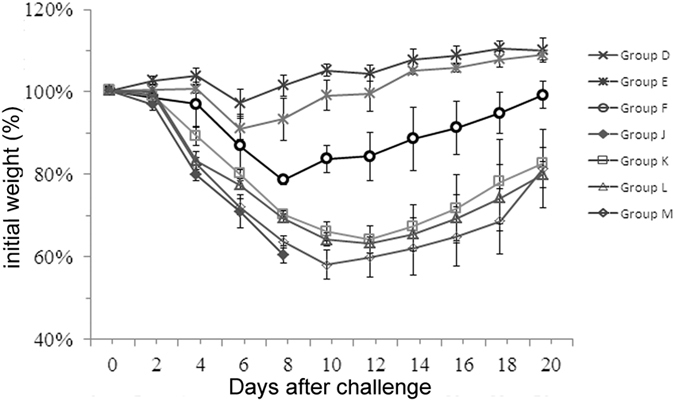
Duration of protective effects afforded by chitosan against H7N9 influenza virus. Mice were dosed twice with 100 μg chitosan (i.n.) in group D–F and K–M, with naïve mice were used as the control group (group J). The mice were then challenged 1, 3, 5, 7, 10, 14 days after the second chitosan administration respectively with a lethal dose of A/Shanghai/2/2013(H7N9) (10×LD_50_). These mice were observed for 21 days. The mice were grouped in the same way as presented in Table 2.

Having observed that the dose of 100 μg of chitosan provided the best protection, we next investigated how long the protective effects of chitosan could last. To this end, we started to challenge the mice on days 1, 3, 5, 7, 10 or14 after the second dose and subsequently monitored the survival rates of these animals. As shown in Table 3, 100% protection of the animals could be achieved even if the lethal virus challenge was conducted 7 days later (Groups D–F, Group K, Table 3) while 80% of the animals were protected 10 days after the second injection (Group L, Table 3), with the survival rates declining to 30% two weeks later (Group M, Table 3). These survival data were consistent with those obtained from determination of the virus loads in the lung tissues (Table 3) and body weight measurements (Fig. 3). These data suggest that the protective immune responses against H7N9 could last as long as 10 days.

**Figure 4:**
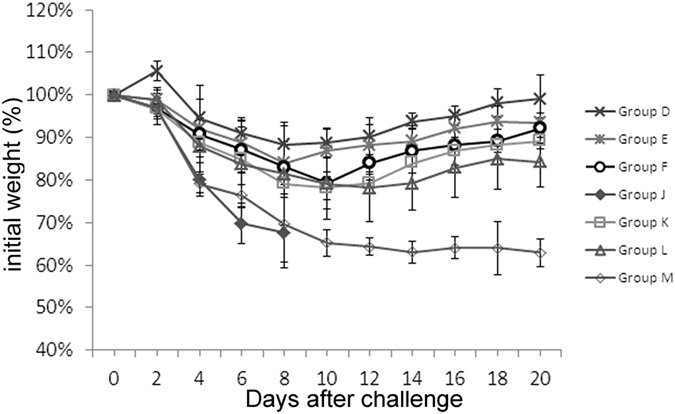
Duration of protective effects afforded by chitosan against PR8 influenza virus. Mice were dosed twice with 100 μg chitosan. The mice were then challenged with a lethal dose of A/Puerto Rico/8/34 (H1N1) (10×LD_50_) on different days to determine the duration of the protective effect. These mice were observed for 21 days. The mice were grouped in the same way as presented in Table 1.

We next determined the duration of the innate immune response induced by chitosan against PR8 virus. After chitosan administration, 100% and 80% protection rates were obtained if the animals were challenged on day 3 and day 5, respectively while 60% of the animals survived even if they were challenged with the virus 10 days later (Table 3). These survival data are largely in agreement with those obtained with the measurements of the body weight (Fig. 4). The slightly less effective protection afforded by chitosan against PR8 compared to that provided by H7N9 remains to be fully determined but could be partially ascribed to different degrees of adaption of the virus growth in mice.

### Full protection of animals from A/California/7/2009 (H1N1) and A/Chicken/Jiangsu/7/2002 (H9N2)

To explore the universality of chitosan-induced protective effects against influenza virus, we employed two more virus strains, NYMC X-179A (H1N1) and A/Chicken/Jiangsu/7/2002 (H9N2), in the challenge studies. We found that all of the animals were protected when the 10 × LD_50_ of 2009 H1N1 or H9N2 viral challenges were carried out 3 days after the second chitosan treatment (Table 4). Collectively, these data provide strong evidence that i.n. administration of chitosan can induce effective immune protection against diverse strains of influenza viruses, demonstrating the universal value of this agent as a prophylactic antiviral agent.

### Intranasal chitosan administration substantially promoted infiltration of leukocytes and increased expression of inflammatory cytokines in the trachea/lung tissues

**Figure 5:**
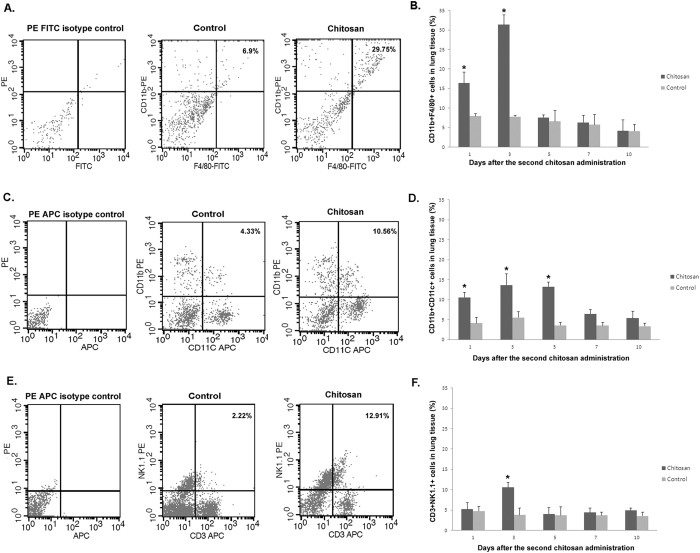
The detection of the phenotype of lung leukocytes by flow cytometry. Mice were given 100 μg chitosan twice. Panels A, C and E were the flow chart of CD11b^+^ F4/80^+^, CD11b^+^ CD11c^+^ and CD3^+^ NK1.1^+^ in leukocytes 3 days after the second chitosan administration, while panel B, D and F were the corresponding histogram on days 1, 3, 5, 7, 10 after the second chitosan administration. The experiments have been repeated two times with the results being expressed as the mean ± SEM of tested mice (n = 5 for each group). * designates significant difference compared to the untreated control group (p < 0.05).

To better understand the mechanism, the entire lung tissue was subject to flow cytometry analysis. Specifically, the cell types in the entire lung tissue were analyzed by using PE-labeled anti-CD11b mAb and FITC-labeled anti-F4/80 mAb to determine the level of macrophages, PE-labeled anti-CD11b mAb and APC-labeled anti-CD11c mAb for dendritic cells (DC), and PE-labeled anti-NK1.1 mAb and APC-labeled anti-CD3 mAb for natural killer cells (NK). We found that the levels of macrophages, DC and NK cells in chitosan treatment group were elevated compared with that in the control group on days 1 to 10 after the second chitosan administration (Fig. 5). Moreover, the level of macrophages in the chitosan group were significantly higher than that in the control group on days 1 and 3 after the second chitosan administration (p value were 0.0264, and 0.0027 respectively) (Fig. 5A,B); moreover, the level of DC cells in chitosan group were significantly higher than that in the control group on days 1,3 and 5 after the second chitosan administration (p value were 0.0208, 0.0334 and 0.0053, respectively) (Fig. 5C,D), while the level of NK cells in chitosan group were significantly higher than that in the control group on day 3 after the second chitosan administration (p value was 0.0212) (Fig. 5E,F). The results suggest that macrophages, DC and NK cells may play important roles in protecting the animal from influenza viral infection.

**Figure 6:**
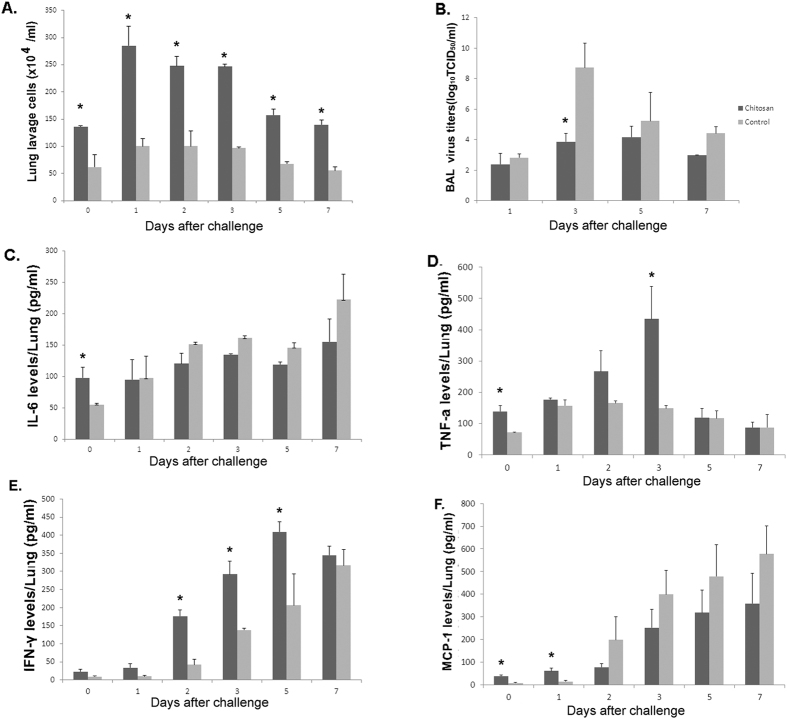
Intranasal chitosan administration upregulated expression of inflammatory cytokines in the lungs. Mice were given 100 μg chitosan twice. Pulmonary cells and lavages were collected as described in the Materials and Methods. Three days after the second chitosan administration, the mice were challenged with a lethal dose of A/Shanghai/2/2013(H7N9) (10 × LD_50_). The number of leukocytes, the virus titers of bronchoalveolar lavage, and selected cytokines were quantitatively analyzed. Panel A depicts the enumeration of pulmonary leukocytes; Panel B depicts the virus titers of bronchoalveolar lavage; Panels C, D, E and F represent data obtained from measurements of IL-6, TNF-α, IFN-γ and MCP-1, respectively. The experiments have been repeated two times with results being expressed as the mean ± SEM of tested mice (n = 3 for each group). *Denotes significant difference between the treatment groups and the untreated control group (p < 0.05).

We also analyzed the infiltration of leukocytes, the virus titers of bronchoalveolar lavage, and the level of cytokines in the BAL derived from mice treated with chitosan. Three days after the second chitosan administration, the mice were challenged with a lethal dose of H7N9 (10×LD_50_) , with the BAL harvested from three mice in each group on days 0, 1, 2, 3, 5, or 7 after challenge. We found that after the second chitosan administration, the number of leukocytes in the lungs of chitosan group was significantly higher than that in the control group (p value were 0.0229, 0.0103, 0.0123, 0.0003, 0.0038 and 0.0042, respectively), both before or after the challenge (Fig. 6A). After challenge, the virus titers of bronchoalveolar lavage in chitosan group was less than that in the control group, particularly three days after (p value was 0.0279) (Fig. 6B). Furthermore, three days after the second chitosan administration, the increased infiltration of leukocytes in the lung lavage were found to be associated with elevated levels of TNF-α, IL-6 and MCP-1 compared to the untreated group. The levels of IL-6, TNF-α and MCP-1 were significantly higher in the lungs of treated mice than those in the control group pre-challenge (p value were 0.0362, 0.0200 and 0.0074, respectively) (Fig. 6C,D,F). Specifically, the levels of IL6, TNF-α, INF-γ and MCP-1 of chitosan group increased after challenge (Fig. 6). The levels of TNF-α in chitosan group were significantly higher than that in the control group on day 3 after challenge (p value was 0.0309) while the levels of INF-γin chitosan group were significantly higher than that in the control group on days 2, 3 and 5 after challenge (p value were 0.0068, 0.0135 and 0.0437, respectively). In addition, the levels of MCP-1 in chitosan group were significantly higher than that in the control group on day 1 after challenge (p value was 0.0181). These data suggest that i.n. administration of chitosan can substantially activate innate immune cells, resulting in increased secretion of cytokines, which is known to possess strong antiviral properties.

## Discussion

Influenza vaccine is one of the most effective measures for prevention of influenza virus infection. Current influenza vaccine mainly consists of the hemagglutinin (HA) and neuraminidase (NA) components of the influenza virus^1^,^24^. However, the two surface glycoproteins mutate frequently in an unpredictable manner, making it necessary to update the virus strains annually^25^,^26^,^27^. In addition, the current manufacturing process for influenza vaccine is time-consuming, which is undesirable in the event of a flu pandemic^28^,^29^. During the epidemic of a newly emerging influenza virus, or the early phase of a major influenza virus outbreak^30^,^31^ (such as human infection of H7N9 avian influenza virus that occurred in China in 2013^3^), the strain-specific vaccines may not yet be available and antiviral drugs do not offer reliable therapeutic effect, it is thus of critical importance to explore alternative approaches.

It is well known that innate immunity is the first line of defense against infectious agents^32^,^33^. Activation of innate immunity through administration of certain cytokines or agents can enhance the host defense against influenza. For example, treatment of mice with low dose exogenous recombinant IFN-α has been shown to prevent influenza virus infection^34^. Lau *et al*. found that the ligand of TLR3, PIKA (double-stranded RNA), could stimulate innate immunity through upregulating CD80 and CD86 on surface of the antigen-presenting dendritic cells, thus inhibiting virus replication^35^.

In this study, we investigate whether i.n. administration of chitosan, a mucosal immunomodulator, could protect BALB/c mice from lethal challenges by H7N9, a highly pathogenic virus. At this time, no H7N9 vaccine has yet been approved. We found that i.n. administration of chitosan could completely protect mice from challenge by wild-type H7N9 virus. The activated innate immune responses by chitosan last for at least 10 days, as demonstrated by the observation that 80% of the mice were still protected 10 days after the chitosan treatment. We verified that the protection is mediated by chitosan, not the vehicle used to dissolve the agent. Specifically, when various amounts of chitosan used in the study were dissolved in the same volume of NaAc solution, we observed that the mice were protected by chitosan from H7N9 challenge in a dose-dependent manner while no protection was afforded by the vehicle NaAc. In our animal protection studies, PR8 was also used as a challenging virus. The protection against PR8 virus seems to be slightly less effective than the protection against H7N9 virus, an observation which we could not fully explain at this time. However, the degree of adaptation of the two viruses to growth in mice could be partially ascribed to the observed difference in chitosan-induced protection. It is of note, however, the results obtained in the PR8 experiments are overall very similar to those obtained from H7N9, i.e., two doses of chitosan at 100 μg could fully protect 80–100% of the animals if the viral challenges were performed 1–5 days after the second injection. Clearly, the i.n route of administration is necessary to ensure full protection. Moreover, we also observed two intranasal injections of chitosan completely protect mice challenged with lethal doses of two different virus strains (H1N1 and H9N2). Taken together, these data provide strong evidence that chitosan can enhance broad host defense against influenza viruses.

Chitosan, a deacetylated product of chitin, is non-toxic, non-irritable, non-antigenic, bioadhesive, biocompatible and biodegradable^36^,^37^. It is of note that the US FDA already approves the use of chitosan in drugs and food. Purified chitosan has been reported to be of low toxicity but possess strong immunomodulatory activity. Previous studies found that nasal administration of chitosan as a vaccine adjuvant can enhance the humoral and cellular immune responses of mice and guinea pigs against influenza, pertussis, diphtheria and tetanus^22^,^23^,^38^. We previously reported that chitosan can function as an adjuvant to improve the immune protection induced by M1- and M2-based candidate vaccines^22^,^23^.

In this study, we found that three days after the second intranasal chitosan administration, the number of leukocytes in the lungs of mice was significantly higher (p < 0.05) in the chitosan group than that in the control group, consistent with observations made by others^39^. Moreover, we also observed significantly higher (p < 0.05) levels of TNF-α in the BAL of the chitosan-treated mice than that from the control mice before challenge. TNF-α is believed to play an important role in chitosan induction of IFN-γ in mice, while IFN-γ produced by natural killer (NK) cells and activated T cells can activate cellular immunity against intracellular pathogens, including viruses^40^. The increased levels of TNF-α and IFN-γ observed in our studies were in agreement with observations made by other investigators who found that chitosan and its derivatives could upregulate cytokines (TNF-α and IL-1) in alveolar macrophages^41^. Furthermore, in the chitosan-treated mice, the levels of MCP-1 and IL-6 in the lungs of chitosan-treated mice were significantly higher than those in the control group before challenge (p < 0.05) (Fig. 6). MCP-1 is one of the key chemokines that regulate the migration and infiltration of monocytes/macrophages^42^. Infiltration and activation of these cells at the site of infection is critical for inhibition of virus replication because these cells can be quickly mobilized to engulf apoptotic, virus-infected cells. Indeed, in virus-infected mice, pulmonary macrophages have been found to have enhanced phagocytic ability^43^,^44^,^45^. In this study, we found that after the second chitosan administration, the level of macrophages, DC and NK cells in chitosan group was higher than that in the control group by flow cytometry, suggesting that macrophages, DC and NK cells, may play important roles in the cleaning process of the influenza virus. Moreover, after chitosan administration, the level of leukocytes cells in NaAc control group was similar to the untreated control group, as determined by flow cytometry analysis. Collectively, these data indicate that the protection was mediated by chitosan.

Thus, the study showed that intranasal administration of chitosan could induce broad immune responses against lethal challenges of H7N9 influenza virus. This could be a viable immunoprophylactic approach. Given the replication of diverse strains of influenza virus that have been inhibited by chitosan, this agent— either alone or in combination with other immune-modulators—could be a valuable universal anti-influenza agent, particularly when strain-specific vaccines are not yet available.

## Materials and Methods

### Viruses, mice and agent

Influenza viruses used in this study included mouse-adapted A/Shanghai/2/2013(H7N9), A/Puerto Rico/8/34 (H1N1), A*/*California*/*7*/*2009 NYMC X-179A (H1N1) and A/Chicken/Jiangsu/7/2002 (H9N2) influenza viruses^22^,^23^,^46^,^47^,^48^,^49^. Influenza A/Shanghai/2/2013(H7N9) virus was isolated from the confirmed H7N9 influenza case-patients in Shanghai, China. The virus strain was provided by Shanghai Public Health Clinical Center, and stored at −70 °C in Biosafety Level-3 (BSL3) Laboratory (Fudan University). To prepare mouse-adapted virus, lung-to-lung passages were performed in BALB/C mice as described in our previous studies^46^,^47^,^48^,^49^. First, the mice were infected with 20 μl of influenza H7N9 viral suspension by the i.n. route. Then, the mice were sacrificed, with the virus in their trachea/lung collected and used for infecting the next batch of mice on the 3rd day after infection. The lung-to-lung passages were repeated three times. After being passaged and adapted in mouse, the viruses were aliquoted and stored at −70 °C until use. The 50% mouse lethal dose (MLD_50_) of the virus was determined (1.55 log_10_TCID_50_/ml) using the Reed-Muench method^50^. In addition, the 50% mouse lethal dose (MLD_50_) of the mouse-adapted A/Puerto Rico/8/34 (H1N1) (1.15 log_10_TCID_50_/ml), A*/*California*/*7*/*2009 NYMC X-179A (H1N1) (3.61 log_10_TCID_50_/ml) and A/Chicken/Jiangsu/7/2002 (H9N2) (1.79 log_10_TCID_50_/ml) influenza viruses were also determined.

Specific-pathogen-free female BALB/c mice (6–8 weeks old) were purchased from Shanghai Laboratory Animal Center, China. All mice were bred in BSL3 or the Animal Resource Center at SIBP in specific-pathogen-free conditions. All experiments involving animals were approved by Animal Care Committee of Fudan University and SIBP in accordance with the animal ethics guidelines of the Chinese National Health and Medical Research Council (NHMRC).

Chitosan was purchased from Sigma (St Louis, MO, USA). A 0.4% (w/v) solution of chitosan was prepared in sodium acetate solution (pH 5.0) at a concentration of 25 mmol/L^22^,^23^. To prepare different doses of chitosan, various amounts of chitosan were dissolved in the same volume of NaAc solution.

### Administration of chitosan and viral challenge in mice

Female BALB/c mice (6–8 weeks old) were administered with various amounts of chitosan i.n. or i.p. , with naïve mice as a negative control (Table 1). Mice were i.n. challenged with lethal doses of 10 × LD_50_ of A/Shanghai/2/2013(H7N9), A/Puerto Rico/8/34 (H1N1), NYMC X-179A (H1N1) or A/Chicken/Jiangsu/7/2002 (H9N2) under sodium pentobarbital anesthesia (60 mg/kg). Mice were weighed for 21 days and checked daily in order to monitor weight loss; animal survival was also observed for 21 days. The original health checks were conducted once a day. When we noticed a rise in mortality, we observed the animals 2–3 times a day; mice were humanely euthanized via cervical dislocation with chloroform (inhalation excess) if weight loss was >30% and if the mice also showed severe clinical signs, such as erratic gait and pre-comatose. At the end of all the experiments, other mice were also humanely euthanized as described above.

For collection of bronchoalveolar lavage (BAL), three mice from each group were randomly selected. The mice were humanely euthanized with chloroform and then bled from the heart with a syringe. Subsequently, the trachea and lung tissues was taken out and washed thrice with a total of 2 ml of PBS containing 0.1% BSA. Following centrifugation for 30 min at 1000 rpm on a bench-top centrifuge to remove cellular debris, the BAL was collected and frozen until use for virus titration.

### Virus titration

To determine the viral loads in the mice, the amounts of the virus in the trachea/lung were determined as described previously^47^,^48^,^49^. In brief, the BAL was serially diluted 10-fold. The diluted BALs were then added to Madin Darby canine kidney (MDCK) cell cultures, which were incubated at 37 °C for 72 h. The virus titer in each group, expressed as 50% tissue culture infection dose (TCID_50_), was calculated by the Reed-Muench method^50^. The virus titer data were represented as the means ± SD in Table 2, 3, 4, or represented as the means ± SEM in Fig. 6.

### Measurement of BAL leukocytes

Three days after the second chitosan administration, the mice were challenged with a lethal dose of H7N9 (10×LD_50_).The BAL harvested from three mice in each group on days 0, 1, 2, 3, 5 or 7 after challenge were centrifuged for 10 min at 200 × g to collect the cells in the lavage. After centrifugation, erythrocytes in the cell pellets were lysed using M-Lyse buffer (R&D Systems, Minneapolis, MN). The cells were washed again with PBS and then resuspended in 1 ml of 0.9% NaCl prior to enumeration with the hemocytometer.

### Analysis of lung leukocytes on flow cytometry

Lung homogenate samples were collected from five mice in each group on various days after the second chitosan administration (see Results). The leukocytes from lung homogenates were detected using PE-labeled anti-CD11b mAb (Biolegend, USA), FITC-labeled anti-F4/80 mAb (Biolegend, USA), APC-labeled anti-CD3 mAb (Biolegend, USA), APC-labeled anti-CD11c mAb (Biolegend, USA), PE-labeled anti-NK1.1 mAb (Biolegend, USA), PE-labeled anti-IgG1 isotype control (BD, USA), APC-labeled anti-IgG1 isotype control (BD, USA) and FITC-labeled anti-IgG1 isotype control (BD, USA) as specified in Results. The stained cells were analyzed on flow cytometry (FACSCalibur, BD, USA) with analyses using CellQuest pro (BD, USA).

### Analyses of lung cytokines

The BAL samples were analyzed for levels of IFN-γ, TNF-α, IL-6 and MCP-1 by ELISA using kit assays obtained from R&D Systems (Minneapolis, MN). The absorbance reading was conducted at a wavelength of 450 nm using a microplate reader. The amounts of the cytokines in the tested samples were determined by a standard curve, with values expressed as the means ± SEM.

### Statistics

The results of test groups were evaluated by one-way ANOVA with the Tukey multiple comparison. The survival rates of the mice in test and control groups were compared using Kaplan Meier survival analysis. If P-value was less than 0.05, the difference was considered significant.

## Additional Information

**How to cite this article**: Zheng, M. *et al*. Intranasal Administration of Chitosan Against Influenza A (H7N9) Virus Infection in a Mouse Model. *Sci. Rep*. **6**, 28729; doi: 10.1038/srep28729 (2016).

## Figures and Tables

**Table 1:** Experimental groups and procedure^a^.

Group	Dose of Chitosan	Route of administration	Times of administration	Days between challenge and the last chitosan administration	Days and Times of chitosan administration before challenge																
					−16	−15	−14	−13	−12	−11	−10	−9	−8	−7	−6	−5	−4	−3	−2	−1	0
A	100 μg	i.n.	1	1																+	Challenge with H7N9 or PR8 virus
B	100 μg	i.n.	1	3														+			
C	100 μg	i.n.	1	5												+					
D	100 μg	i.n.	2	1														+		+	
E	100 μg	i.n.	2	3												+		+			
F	100 μg	i.n.	2	5										+		+					
G	30 μg	i.n.	2	3												+		+			
H	10 μg	i.n.	2	3												+		+			
I	100 μg	i.p.	2	3												+		+			
J	control	−	−	−																	
K	100 μg	i.n.	2	7								+		+							
L	100 μg	i.n.	2	10					+		+										
M	100 μg	i.n.	2	14	+		+														

^a^Mice were treated with various doses of chitosan and challenged with influenza viruses. Specifically, the animals in groups A–C were given 100 μg chitosan once intranasally (i.n.) and subsequently challenged with the virus on 1, 3 or 5 days following the chitosan treatment. In groups D–F and K–M, the animals were dosed twice with 100 μg chitosan (i.n.) and then challenged on 1, 3, 5, 7, 10 or 14 days following the second chitosan administration. In groups G and H, the mice were dosed twice with 30 or 10 μg chitosan (i.n) and then challenged 3 days after the second chitosan administration. In group I, the animals were dosed twice with 100 μg chitosan via the intraperitoneal route (i.p.) and were challenged 3 days after the second chitosan administration. The naïve mice were used as the control group (group J). In all viral challenge groups, the mice were infected with a lethal dose of A/Shanghai/2/2013(H7N9) (10×LD_50_) or A/Puerto Rico/8/34 (H1N1) (10×LD_50_) to determine the protective effect; the animals were observed for 21 days. + The mice were administrated with various dose of chitosan.

**Table 2:** Chitosan affords protection against H7N9 or PR8 infection^a^.

Group	Dose of Chitosan	Route of administration			Protection against H7N9 virus challenge			Protection against PR8 virus challenge		
			Times of administration	Days between challenge and the last chitosan administration	Lung virus titers (log_10_TCID_50_/ml)^b^	Weight change (%)^b^^,^^c^	No. of survivors/no. tested	Lung virus titers (log_10_TCID_50_/ml)^b^	Weight change (%)^b^^,^^c^	No. of survivors/no. tested
A	100 μg	i.n.	1	1	3.37 ± 0.52*^,^^i^^,^^j^^,^^k^^,^^l^	4.24 ± 10.14*^,^^f^^,^^i^^,^^j^^,^^k^^,^^l^	10/10*^,^^k^^,^^l^	6.33 ± 1.15*^,^^g^^,^^h^^,^^i^^,^^j^	17.13 ± 3.50*^,^^l^	6/10*^,^^k^^,^^l^
B	100 μg	i.n.	1	3	4. 75 ± 0.89	6.05 ± 5.83*^,^^f^^,^^i^^,^^k^^,^^l^	10/10*	5.67 ± 0.64*^,^^h^^,^^l^	15.75 ± 4.26*^,^^l^	8/10*^,^^k^^,^^l^
C	100 μg	i.n.	1	5	5.00 ± 0.93^d^^,^^g^	19.26 ± 2.83*^,^^d^^,^^e^^,^^g^^,^^h^^,^^k^^,^^l^	10/10*^,^^k^^,^^l^	5.73 ± 1.15*^,^^h^^,^^j^^,^^l^	12.25 ± 2.38*^,^^k^^,^^l^	7/10*^,^^k^^,^^l^
D	100 μg	i.n.	2	1	3.38 ± 0.52*^,^^i^^,^^i^^,^^k^^,^^l^	2.91 ± 2.21*^,^^h^^,^^i^^,^^j^^,^^k^^,^^l^	10/10*^,^^k^^,^^l^	4.75 ± 0.46*^,^^d^^,^^k^^,^^l^	10.11 ± 3.48*^,^^j^^,^^k^^,^^l^	9/10*^,^^k^^,^^l^
E	100 μg	i.n.	2	3	4.75 ± 1.04	10.81 ± 5.21*^,^^g^^,^^i^^,^^k^^,^^l^	10/10*^,^^k^^,^^l^	4.25 ± 0.46*^,^^d^^,^^e^^,^^f^^,^^k^^,^^l^	13.05 ± 3.86*^,^^k^^,^^l^	10/10*^,^^k^^,^^l^
F	100 μg	i.n.	2	5	4.87 ± 0.90^d^^,^^g^	18.69 ± 5.27*^,^^d^^,^^e^^,^^g^^,^^h^^,^^k^^,^^l^	10/10*^,^^k^^,^^l^	5.13 ± 0.35*^,^^d^^,^^k^	15.29 ± 5.03*^,^^l^	8/10*^,^^k^^,^^l^
G	30 μg	i.n.	2	3	5.08 ± 1.51^d^^,^^g^	12.16 ± 4.91*^,^^d^^,^^g^^,^^k^^,^^l^	8/10*^,^^k^^,^^l^	4.67 ± 0.58*^,^^d^^,^^f^^,^^k^^,^^l^	19.25 ± 3.94*^,^^g^^,^^l^	9/10*^,^^k^^,^^l^
H	10 μg	i.n.	2	3	5.19 ± 1.13^d^^,^^g^	28.45 ± 5.04^d^^,^^e^^,^^f^^,^^g^^,^^h^^,^^i^^,^^j^^,^^l^	3/10*^,^^d^^,^^e^^,^^f^^,^^g^^,^^h^^,^^i^^,^^j^	6.13 ± 0.35*^,^^g^^,^^h^^,^^j^^,^^l^	21.29 ± 4.35*^,^^f^^,^^g^^,^^h^	6/10*^,^^k^^,^^l^
I	100 μg	i.p.	2	3	5.38 ± 1.51^d^^,^^g^	37.37 ± 1.04^d^^,^^e^^,^^f^^,^^g^^,^^h^^,^^i^^,^^j^^,^^k^	1/10*^,^^d^^,^^e^^,^^f^^,^^g^^,^^h^^,^^i^^,^^j^	7.33 ± 0.35,^e^^,^^f^^,^^g^^,^^h^^,^^I^^,^^j^^,^^k^	27.54 ± 4.81^d^^,^^e^^,^^f^^,^^g^^,^^h^^,^^i^^,^^j^	0/10^d^^,^^e^^,^^f^^,^^g^^,^^h^^,^^i^^,^^j^^,^^k^
J	control	–	–	–	6.63 ± 1.60^d^^,^^e^^,^^f^^,^^g^^,^^h^	38.02 ± 0.88^d^^,^^e^^,^^f^^,^^g^^,^^h^^,^^i^^,^^j^	0/10^d^^,^^e^^,^^f^^,^^g^^,^^h^^,^^i^^,^^j^^,^^k^^,^^l^	7.75 ± 0.46 d^,^^e^^,^^f^^,^^g^^,^^h^^,^^i^^,^^j^^,^^k^	31.59 ± 3.76^d^^,^^e^^,^^f^^,^^g^^,^^h^^,^^i^^,^^j^^,^^k^	0/10^d^^,^^e^^,^^f^^,^^g^^,^^h^^,^^i^^,^^j^^,^^k^^,^^l^

^a^Mice were treated with chitosan as described in the table. After the last administration, mice were challenged with a lethal dose (10 × LD_50_) of influenza A/Shanghai/2/2013(H7N9) or A/Puerto Rico/8/34 (H1N1) virus. Bronchoalveolar lavages (BAL) were collected 3 days post-infection for virus titration in the lungs while the survival rates of mice were obtained during the 21-day observation period.

^b^Results are expressed as the mean ± SD of tested mice in each group.

^c^Represent the percentage of bodyweight loss in animals after viral challenge compared with that observed before the challenge. The data were obtained from mice on day 7 after the viral challenge.

^d^Denotes significant difference compared to the group A (*P* < 0.05).

^e^Denotes significant difference compared to the group B (*P* < 0.05).

^f^Denotes significant difference compared to the group C (*P* < 0.05).

^g^Denotes significant difference compared to the group D (*P* < 0.05).

^h^Denotes significant difference compared to the group E (*P* < 0.05).

^i^Denotes significant difference compared to the group F (*P* < 0.05).

^j^Denotes significant difference compared to the group G (*P* < 0.05).

^k^Denotes significant difference compared to the group H (*P* < 0.05).

^l^Denotes significant difference compared to the group I (*P* < 0.05).

*Significantly different from the control groups (group J) (P < 0.05).

**Table 3:** Duration of protection against H7N9 or PR8^a^.

Group	Dose of Chitosan	Route of administration	Times of administration	Days between challenge and the last chitosan administration	Protection against H7N9 virus			Protection against PR8 virus		
					Lung virus titers (log_10_TCID_50_/ml)^b^	Weight change (%)^b^^,^^c^	Survival (survivors/total)	Lung virus titers (log_10_TCID_50_/ml)^b^	Weight change (%)^b^^,^^c^	Survival (survivors/ total)
D	100 μg	i.n.	2	1	3.78 ± 0.97*	5.06 ± 5.05*^,^^e^^,^^f^^,^^g^^,^^h^^,^^i^	10/10*^,^^i^	4.75 ± 0.46*^,^^g^^,^^h^^,^^i^	10.11 ± 3.48*,^g^^,^^i^	9/10*,^h^^,^^i^
E	100 μg	i.n.	2	3	3.83 ± 0.75*	12.28 ± 2.79*^,^^d^^,^^f^^,^^g^^,^^h^^,^^i^	10/10*^,^^i^	4.25 ± 0.46*^,^^g^^,^^h^^,^^i^	13.05 ± 3.86*,^i^	10/10*,^h^^,^^i^
F	100 μg	i.n.	2	5	4.89 ± 1.17*	19.51 ± 8.05*^,^^d^^,^^e^^,^^i^	10/10*^,^^i^	5.13 ± 0.35*^,^^g^^,^^h^^,^^i^	15.29 ± 5.03*,^i^	8/10*,^h^^,^^i^
K	100 μg	i.n.	2	7	4.33 ± 0.52*	24.65 ± 1.17*^,^^d^^,^^e^	10/10*^,^^i^	6.33 ± 0.45*^,^^d^^,^^e^^,^^f^	18.82 ± 3.22*,^d^^,^^i^	7/10*
L	100 μg	i.n.	2	10	4.33 ± 1.03*	26.01 ± 1.28*^,^^d^^,^^e^	8/10*^,^^i^	6.43 ± 0.15*,^d^^,^^e^^,^^f^	17.46 ± 2.27*,^i^	6/10*
M	100 μg	i.n.	2	14	5.67 ± 1.32	30.44 ± 1.30^d^^,^^e^^,^^f^	3/10*^,^^d^^,^^e^^,^^f^^,^^g^^,^^h^	7.03 ± 0.52,^d^^,^^e^^,^^f^	27.06 ± 2.35^d^^,^^e^^,^^f^^,^^g^^,^^h^	2/10*,^d^^,^^e^^,^^f^
J	control	–	–	–	7.11 ± 2.93	36.64 ± 2.05^d^^,^^e^^,^^f^^,^^g^^,^^h^	0/10^,^^d^^,^^e^^,^^f^^,^^g^^,^^h^^,^^i^	7.75 ± 0.46 d^,^^e^^,^^f^^,^^g^^,^^h^	31.59 ± 3.76 d^,^^e^^,^^f^^,^^g^^,^^h^	0/10 d^,^^e^^,^^f^^,^^g^^,^^h^^,^^i^

^a^Mice were treated with chitosan as described above. After the second chitosan administration, the mice were challenged with a lethal dose (10 × LD_50_) of influenza A/Shanghai/2/2013(H7N9) or A/Puerto Rico/8/34 (H1N1) virus. Bronchoalveolar lavages (BAL) were collected 3 days post-infection for virus titration in the lungs while the survival rates of mice were obtained during the 21-day observation period.

^b^Results are expressed as the mean ± SD of tested mice in each group.

^c^Represent the percentage of bodyweight loss in animals after viral challenge compared with that observed before the challenge. The data were obtained from mice on day 7 after the viral challenge.

^d^Denotes significant difference compared to the group D (*P* < 0.05).

^e^Denotes significant difference compared to the group E (*P* < 0.05).

^f^Denotes significant difference compared to the group F (*P* < 0.05).

^g^Denotes significant difference compared to the group K (*P* < 0.05).

^h^Denotes significant difference compared to the group L (*P* < 0.05).

^i^Denotes significant difference compared to the group M (*P* < 0.05).

^*^Significant difference from the control groups (P < 0.05).

**Table 4:** Protection of animals by chitosan against 2009 H1N1 and H9N2^a^.

Group	*Administration Route*	Number of injection	Days between chitosan injection and viral challenge	Challenge virus	Protection against various virus challenge	
					Weight change (%)^b^^,^^c^	Survival rate
Chitosan control	*i.n*.	2	3	A/California/7/2009 (H1N1)	11.61 ± 3.42*	10/10*
	–	–	–		32.13 ± 2.26	0/10
Chitosan control	*i.n*.	2	3	A/Chicken/Jiangsu/7/2002 (H9N2)	13.3 ± 4.04*	10/10*
	–	–	–		38.2 ± 4.87	0/10

^a^Mice were administered with 100 μg chitosan twice. After the last administration, mice were challenged with a lethal dose (10 × LD_50_) of A/California/7/2009 (H1N1), or A/Chicken/Jiangsu/7/2002 (H9N2) influenza virus, respectively. The survival rates were obtained by monitoring the animals for 21 days.

^b^Results are expressed as the means ± SD in each group.

^c^Represents the percentage of bodyweight loss in animals after viral challenge compared with that observed before the challenge. The data were obtained from mice on day 7 after the viral challenge.

^*^Denotes significantly difference from the control groups (P < 0.05).
